# Institutional delivery in rural India: the relative importance of accessibility and economic status

**DOI:** 10.1186/1471-2393-10-30

**Published:** 2010-06-06

**Authors:** Amy J Kesterton, John Cleland, Andy Sloggett, Carine Ronsmans

**Affiliations:** 1 London School of Hygiene and Tropical Medicine, 50 Bedford Square, London, WC1B 3DP, UK

## Abstract

**Background:**

Skilled attendance at delivery is an important indicator in monitoring progress towards Millennium Development Goal 5 to reduce the maternal mortality ratio by three quarters between 1990 and 2015. In addition to professional attention, it is important that mothers deliver their babies in an appropriate setting, where life saving equipment and hygienic conditions can also help reduce the risk of complications that may cause death or illness to mother and child. Over the past decade interest has grown in examining influences on care-seeking behavior and this study investigates the determinants of place of delivery in rural India, with a particular focus on assessing the relative importance of community access and economic status.

**Methods:**

A descriptive analysis of trends in place of delivery using data from two national representative sample surveys in 1992 and 1998 is followed by a two-level (child/mother and community) random-effects logistical regression model using the second survey to investigate the determinants.

**Results:**

In this investigation of institutional care seeking for child birth in rural India, economic status emerges as a more crucial determinant than access. Economic status is also the strongest influence on the choice between a private-for-profit or public facility amongst institutional births.

**Conclusion:**

Greater availability of obstetric services will not alone solve the problem of low institutional delivery rates. This is particularly true for the use of private-for-profit institutions, in which the distance to services does not have a significant adjusted effect. In the light of these findings a focus on increasing demand for existing services seems the most rational action. In particular, financial constraints need to be addressed, and results support current trials of demand side financing in India.

## Background

Appropriate delivery care is crucial for both maternal and perinatal health and increasing skilled attendance at birth is a central goal of the safe motherhood and child survival movements. Skilled attendance at delivery is an important indicator in monitoring progress towards Millennium Development Goal 5 to reduce the maternal mortality ratio by three quarters between 1990 and 2015 [1]. In addition to professional attention, it is important that mothers deliver their babies in an appropriate setting, where life saving equipment and hygienic conditions can also help reduce the risk of complications that may cause death or illness to mother and child [2].

Over the past decade interest has grown in examining influences on care-seeking behavior. As cited in the "three delays" model, three main inhibitors to health care service utilisation exist: the delay in deciding to seek care, the delay in reaching an adequate health care facility and the delay in receiving adequate care at that facility [3]. The first delay may be due to a lack of understanding of danger signs, the absence of the decision maker from the household, the low status of the woman, cost, previous unsatisfactory experience with the health care system and perceived low quality of care [4]. Phase 2 delays may be due to distance from facility, lack of transportation, difficult terrain and the high cost of travel [3].

Research consistently shows that high cost is an important constraint to service utilization particularly for the poor [5-11]. In India studies show a very high out of pocket expenditure on delivery care, and, although the private sector is more expensive, the cost of public sector inpatient care services has increased since the 1990s [12]. Hence, income is a major determinant of care seeking [13]. Recent analysis of the third National Family Health Survey (2005/6) shows 13% of women in the lowest wealth quintile accessing institutional delivery care compared with 84% in the highest [14]. The importance of proximity to health services as a factor affecting utilization has also been stressed. It exerts a dual influence on health care utilisation. Long distance can be an obstacle to reaching a health facility as well as a disincentive to even try to seek care. Rural populations are particularly disadvantaged as they often lack reliable means of transportation. A sizable proportion of maternal deaths in developing countries occur on the way to hospital; other women are almost beyond help by the time they arrive [15]. Some studies (including in India) have found that geographical access has a greater effect on utilisation than socioeconomic factors [16,17], particularly in rural areas with limited service provision [18,19].

India's Child Survival and Safe Motherhood Programme (CSSM), launched in 1992, involved training of physicians and traditional birth attendants (TBAs), provision of aseptic delivery kits and expansion of existing rural health services to include facilities for institutional delivery i.e. supplying essential equipment to district, sub-district and first level referral facilities to deal with high risk obstetric emergencies (MOHFW 1997-8). The initiative aimed to improve the proportion of pregnant women receiving three antenatal visits, and the proportion of deliveries conducted by trained attendants. The CSSM gave way to the Reproductive and Child Health (RCH) programme in 1997, at which point the scope was widened to include other reproductive and child health services [20]. The second five year phase of the RCH programme (RCH II) is currently being initiated and contains a comprehensive newborn health strategy that includes promotion of institutional deliveries, with cash subsidies for poor families and compensation of TBAs facilitating the process [21]. In areas remote from facilities, improvement of home-based newborn care via auxillary nurse-midwives is envisaged [22,23].

An investigation of the determinants of place of delivery in rural India is the primary objective of this paper. It adds to existing work by using a logistic model with village-level random effect and data for the whole of rural India. Previous studies used standard regression techniques which do not take into account the clustered nature of multi-level data at each level and can give misleading results in terms of both central estimates and their pecision. In particular this paper aims to assess the relative importance of community access and household economic status in determining place of delivery, while also examining the influence of birth order, mother's education and region. A secondary objective is to investigate the choice of provider amongst those who opt for institutional delivery and the impact of these same factors on this decision. Although a few deliveries take place in NGO or charity hospitals, the major choice is between private - for-profit and government providers.

## Methods

The analysis is based on the first two rounds of the National Family Health Survey (NFHS). Approval for the use of this data was gained from the Demographic and Health surveys (DHS) online-archive. Trends in place of delivery between NFHS-1 and 2 are described, followed by two -level modelling using the rural NFHS-2 data. This analysis is based only on rural births because community access was not measured in the urban sample.

### The National Family Health Surveys

The NFHS is a large-scale nationally representative survey, conducted on a sample of ever married women aged 15-49 years in 1992/3 (NFHS-1: n = 98777) and repeated in 1998/9 (NFHS-2: n = 90303). Samples were designed to provide separate estimates for states as a whole and for rural and urban areas. Within each of the sampling domains (rural or urban), systematic, multi-stage stratified sampling was used. In rural areas a first stage selection of primary sampling units (PSUs) using Probability Proportionate to Size was followed by random selection of households from within each PSU to identify women aged 15-49 years. Three separate questionnaires were then administered. The first elicited information regarding the woman's household, the second was addressed to the woman herself and the third collected village level information for rural clusters only. In both NFHS-1 and NFHS-2, certain population strata were over sampled. Weights were applied in the analysis to adjust for unequal selection probabilities.

### Statistical Methods

Firstly, a description of trends in the percentage of births taking place in public or private - for profit facilities between 1989-1998 was carried out. Data on place of delivery are available for rural births in the four years preceding NFHS-1 (1989-92) and in the three years preceding NFHS-2 (1996-98). Values for 1993-5 were estimated using simple linear interpolation.

To investigate the determinants of institutional delivery (Table 1) an analysis was carried out on the 21911 rural births from NFHS-2 (1996-8) with complete information on the covariates. Six hundred and sixty nine cases were dropped (only 3% of the original sample) because of missing data. Secondly, the determinants of choice of private delivery versus public sector delivery were investigated on 5082 institutional births (Table 2).

**Table 1:** The determinants of institutional delivery in rural India: adjusted odds ratios and predicted probabilities from random effects logistic regression model of NFHS 2 data.

	n	% Institutional delivery	Unadjusted OR & 95% CIs	Adjusted OR & 95% CIs	Adjusted Predicted Probability (%)
**Geographical Access**					

**Distance to hospital**	Change in AIC due to removal of distance to hospital from model: +66.3				

31 + km	3027	13.4	1	1	9.7
16-30 km	4544	18.5	**1.98 (1.47-2.69)**	**1.40 (1.09-1.77)**	13.0
6-15 km	8180	23.8	**2.81 (2.13-3.72)**	**1.79 (1.43-2.24)**	16.1
Up to5 km	6160	32.0	**5.08 (3.81-6.78)**	**2.43 (1.93-3.06)**	20.7

**Economic Status**					

**Wealth Quartiles**	Change in AIC due to removal of wealth from model: +361.9				

1 (poorest)	5490	9.5	1	1	9.2
2	5429	15.7	**1.76 (1.56-1.99)**	**1.40 (1.24-1.59)**	12.4
3	5152	24.9	**3.19 (2.81-3.61)**	**1.95 (1.71-2.22)**	16.4
4 (richest)	5840	44.0	**8.44 (7.42-9.62)**	**3.76 (3.26-4.34)**	27.6

**Indicators of preference for place of delivery**					

**Maternal Education**	Change in AIC due to removal of maternal education from model: +376.1				

None	13009	13.0	1	1	11.6
Primary	3540	26.8	**2.32 (2.08-2.59)**	**1.60 (1.43-1.79)**	17.3
Secondary	4435	44.2	**5.60 (5.06-6.20)**	**2.60 (2.33-2.91)**	25.4
Higher	927	67.4	**14.21(11.76-17.17)**	**4.79 (3.90-5.87)**	38.6

**Region**	Change in AIC due to removal of region from model: +685.5				

North	12983	15.6	1	1	9.3
East	4633	22.2	**2.99 (2.47-3.62)**	**2.80 (2.35-3.34)**	22.3
West	1440	34.7	**4.89 (3.95-6.04)**	**3.35 (2.76-4.06)**	25.6
South	2855	58.7	**14.51 (12.15-17.33)**	**8.77 (7.45-10.32)**	47.3

**Birth Order**	Change in AIC due to removal of birth order from model: +475.8				

1	5916	39.4	**2.68 (2.46-2.93)**	**2.41 (2.20-2.64)**	26.9
2-3	9267	23.0	1	1	13.2
4-6	5325	11.9	**0.53 (0.47-0.59)**	**0.80 (0.71-0.90)**	10.9
7 +	1403	9.3	**0.54 (0.44-0.67)**	1.00 (0.80-1.24)	13.2

**rho (P-value)**				**0.27 (P < 0.001)**	

Note. Significant associations (P < 0.05) are shown in bold

Adjusted odds ratios are calculated by adjustment for all other factors in the model, and including random effects

Akaike's Information Criterion (AIC)

**Table 2:** Determinants of choice of a private-for profit institution in institutional deliveries: unadjusted and adjusted odds ratios from logistic regression for rural births (NFHS 2).

Determinants		Private-for profit delivery			
	**n**	**% Private delivery**	**Unadjusted OR & 95% CIs**	**Adjusted OR & 95% CIs**	**Adjusted Predicted Probability (%)**

**Geographical Access**					

**Distance to private and public institutions**	Change in AIC due to removal of distance from model: -3.3				

Both institutions are the same distance	3428	40.3	1	1	30.7
Public institution closer (by > 5 km)	255	41.6	0.92 (0.54-1.07)	1.04 (0.62-1.73)	31.5
Private institution closer (by > 5 km)	1399	49.4	**1.38 (1.07-1.77)**	1.11 (0.87-1.40)	32.9

**Economic Status**					

**Wealth Quartiles**	Change in AIC due to removal of wealth from model: + 85.2				

1 (poorest)	503	30.4	1	1	22.8
2	824	31.4	**1.39 (1.05-1.84)**	1.21 (0.91-1.60)	26.4
3	1240	37.5	**2.07 (1.58-2.72)**	**1.48 (1.12-1.96)**	30.6
4 (richest)	2515	51.7	**4.75 (3.65-6.19)**	**2.97 (2.23-3.96)**	46.8

**Indicators of preference for place of delivery**					

**Maternal Education**	Change in AIC due to removal of maternal education from model: +31.2				

None	1628	36.4	1	1	30.4
Primary	925	35.4	1.05 (0.84-1.32)	0.86 (0.68-1.08)	27.4
Secondary	1921	46.1	**1.79 (1.48-2.16)**	1.17 (0.95-1.45)	33.9
Higher	608	61.0	**4.67 (3.57-6.34)**	**2.17 (1.60-2.95)**	48.7

**Region**	Change in AIC due to removal of region from model: +251.2				

North	1974	43.9	1	1	40.9
East	1004	12.8	**0.10 (0.07-0.14)**	**0.10 (0.07-0.14)**	6.2
West	480	59.8	**1.60 (1.17-2.19)**	**1.43 (1.03-1.98)**	49.7
South	1624	55.2	**1.61 (1.26-2.05)**	**1.52 (1.17-1.97)**	51.2

**Birth Order**	Change in AIC due to removal of birth order from model: +5.1				
1	2098	44.0	**1.26 (1.08-1.46)**	**1.24 (1.06-1.45)**	35.4
2-3	2248	44.0	1	1	30.7
4-6	611	37.3	**0.80 (0.62-1.01)**	0.98 (0.76-1.26)	30.2
7 +	125	29.6	**0.50 (0.30-0.83)**	0.70 (0.41-1.18)	23.6

**Rho (P-value)**				**0.41 (0.000)**	

Note. Significant associations (P < 0.05) are shown in bold

Adjusted odds ratios are calculated by adjustment for all other factors in the model, and including random effects

Akaike's Information Criterion (AIC)

In both analyses, multi-level modelling was applied because births, which represent the first level, are clustered within families at the second level (same mother), and within villages at the third level [24,25]. However, because only births over a three-year period preceding NFHS-2 have been analysed, the majority of mothers contribute only one child to the analysis and comparison of Huber-White standard errors found little effect of clustering at the family level. Therefore a two-level (child/mother and village) random effects logistic regression model was used in all analyses. The random effects procedure used (Stata v 9) assumes normal error for the random component. Using different numbers of quadrature points did not affect the results.

The model outcomes represent the expected or 'true' propensity of institutional delivery for a birth, net of the shared propensity of villagers to follow their neighbours' behaviour. The model provides a set of fixed effects associated with the covariates (acting at the birth and village level) and an unexplained residual term or village level random effect. The latter measures the extent to which the institutional delivery probabilities of children from the same villages resemble each other as compared with this outcome among children in different communities. It can be expressed as the proportion of the total unexplained variance in the outcome that is due to differences between villages, labelled rho in Table 1and 2. Multi-level methods allow assessment of the strength of effect of each covariate and the importance of each level in determining the outcome i.e. how much of the variation found can be credited to the different levels [25]. All first-order interactions were examined but none was found significant. The Akaike's Information Criterion (AIC) was used to assess the relative strength of effect of the different factors. The AIC value reflects the increase in unexplained variation when a factor is omitted from the model [26].

#### Selection and definition of variables

##### Place of delivery

Information on place of delivery was ascertained from mothers for all live births in the three years preceding the survey. The vast majority of institutional births (over 82% in NFHS-II) take place in hospital but a minority are in public community or primary health centres and subcentres. These more basic facilities are available closer to home, but are not primarily designed for delivery. A programme to upgrade primary health centres is now taking place, but it had not begun in 1999, when the Child Survival and Safe Motherhood Program was focusing on first referral level facilities. As births receiving skilled attendance at home are a small sub-group (9.6% of all births), it was decided to combine them with other home deliveries, thus yielding a dichotomous outcome, institutional versus home delivery. The sector of the delivery facility was ascertained from mothers, but the availability of obstetric services was not measured. However, most hospitals are intended for maternity care, typically having comprehensive emergency obstetric care facilities (with surgery) available. As very few institutional births (n = 143) took place in facilities run by non-governmental organisations or charities, these births were omitted from the relevant part of the analysis leaving two categories: public- and private- for profit-sector.

The choice of covariates was based on extensive exploratory analysis and on theoretical considerations. Uptake of maternity services is likely to be determined by mother's education and autonomy, caste, region, birth order and community access, mediated by economic status of the household. Exploratory analysis showed that caste and autonomy were weakly related to the outcomes and were therefore omitted. In the analysis, distance to the nearest hospital represents access and economic status is represented by household wealth. Education is well established as a strong predictor of service use, even after adjusting for income. This effect probably reflects greater identification with allopathic medicine and procedures. Birth order is included because uptake of services usually declines with each succeeding birth and region represents the wide diversity of cultural, political and economic factors in India that shape preferences. India is very heterogeneous but as no first order interactions were found between region and other covariates, it is deemed valid in this circumstance to make regional generalizations. A more complete representation of determinants would have included perceived quality of obstetric services and pregnancy-specific complications but such specific data were not collected in NFHS.

Some of these covariates used in the analysis are self-explanatory (mother's education and birth order of the child). Others are defined below:

##### *Distance to facilities *(public and private-for-profit)

This factor was measured as kilometres from the village centre by NFHS staff who questioned key informants in each cluster. In this analysis distances to the nearest public and private-for-profit sector hospital are considered because they represent the most important location of institutional delivery (>82%). It is important to note that, the closest hospitals are not necessarily those actually used by women in the village. Also, while distance is the best measure of access available it does not reflect other physical barriers which will vary e.g. difficult terrain. The NFHS collected village-level information including the presence of a sealed road but, in exploratory analysis, the latter was found to be unrelated to place of delivery.

##### Wealth index

Wealth quintiles were already constructed on the NFHS data file using assets or wealth information gathered through the NFHS household questionnaire. The standardized asset scores were used to create the break points that define wealth quintiles and the sample was then divided into five population quintiles of equal size [27,28]. Because the rural population is typically poorer than the urban one, the richest two quintiles were underrepresented and therefore, in this analysis were combined to produce four wealth strata of approximately equal size. It would have been possible to re-create quintiles from the raw assets but quartiles were preferred to preserve symmetry with the two other key co-variates - distance and education - that were represented by four categories.

##### Region

Indian States were grouped into the four main geographical regions, North (Bihar, Haryana, Himachal Pradesh, Jammu, Madhya Pradesh, Punjab, Rajasthan, Uttar Pradesh, New Delhi), South (Andhra Pradesh, Karnataka, Kerala, Tamil Nadu, Goa), East (Assam, Orissa, West Bengal, Manipur, Meghalaya, Mizoram, Nagaland, Sikkim (NFHS-2 only), Arunchal Pradesh, Tripura) and West (Gujarat, Maharashtra). The four regions capture in a crude way the wide cultural and other variations of the country and have been used in many other analyses (e.g. [29]).

## Results

### Trends in place of delivery

**Figure 1:**
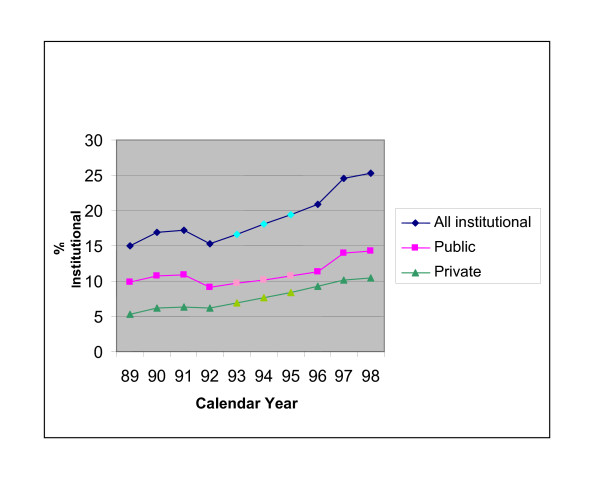
**Trends in percentage of births taking place in a public or private sector facility: rural India 1989-1998 (NFHS I & II)**.

Figure 1 shows the overall prevalence of institutional births in rural India has increased by ten percent (in absolute terms) from about 15% in 1989 to 25% in 1998. Progress has been slightly greater in the private-for-profit than public sector.

**Figure 2:**
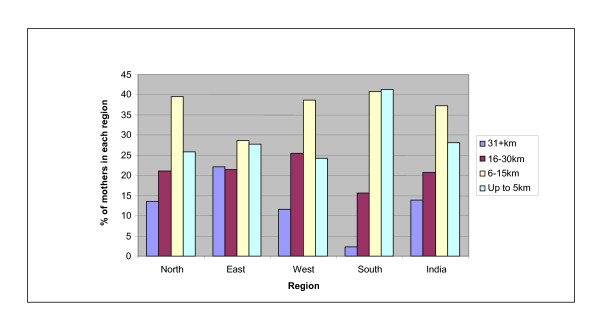
**Distance to the nearest hospital in regions of rural India (NFHS-II)**.

Figure 2 shows the distance to the nearest hospital for rural communities in 1998/9. About one third of rural Indians live within 5 kms of a hospital and nearly two-thirds within 15 kms. The East is most poorly served, while the South has more accessible services with over 40% of the population within 5 kms of an institution.

### Determinants of place of delivery

The determinants of institutional delivery are assessed in Table 1. Strong associations between place of delivery and community access and household wealth are apparent, and education, region and birth order are also all important influences. Among the least privileged households, those with poorest access, wealth and education, only 10-15% of births were delivered in a medical facility. This proportion rises to 32% among households living within 5 km of a hospital, to 44% among the richest households and to 67% among the small minority of households where the mother has tertiary education. The probability of an institutional delivery rises from 9% in high order births to 39% in first births. Huge regional differences are apparent. In the South 59% of births were institutional compared with only 16% in the North. All these associations remain statistically significant after adjustment.

For ease of interpretation, adjusted odds ratios are also expressed as model-based predicted probabilities of institutional delivery. The adjusted results suggest that the influence of household wealth is stronger than that of geographical access. In the poorest 25% of households (the lowest quartile) and in households living 31 kms or more from the nearest hospital, the predicted probability of an institutional delivery is about 10%. In households with good access (<6 kms), the probability rises to 21% but in the richest quartile of the rural population the figure is 28%. A shift in wealth from the poorest to wealthiest group therefore has a greater impact than an equivalent change in accessibility of services. This is a valid comparison because there is a common base of approximately 10% institutional delivery in the least fortunate groups for both household wealth and geographic access, and the most fortunate groups are equivalently favourable, with reasonable numbers in each group.

The greater explanatory power of wealth compared with community access is confirmed by examination of AIC values. The AIC value rises by 362 when wealth is omitted from the model compared with a rise of 66 when access is omitted. The adjusted associations of education and birth order are of the same magnitude as for wealth but region is the strongest covariate, with an AIC value of 686. Regional differentials are indeed strikingly large and the low probability of institutional delivery in the North cannot be explained by disparities of access, wealth or education.

The other highly significant result is the adjusted rho value. In Table 1 the figure of 0.27 (p < 0.001) means that over 25% of variation in institutional delivery care-seeking in rural areas is accounted for by variation at the village level.

The determinants of choice of type of facility - private-for-profit versus public sector - are assessed in Table 2. In this analysis community access is re-defined combining distance to private-for-profit and public sector institutions. Although the highest proportion of private-for-profit deliveries (49%) occur when a private-for profit institution is closer than a public one, this pattern does not remain significant after adjustment. As expected, household wealth emerges as the major determinant of choosing a private-for-profit facility. The proportion of all institutional deliveries that occur in private-for-profit sector medical facilities rises from 30% in poorest households to over 50% in the richest quartile. This association remains strong after adjustment and by comparison, that of mother's education is weak. Only the variable region has a greater individual effect than that of wealth. There is great regional variation in choice, with only 13% of institutional births taking place in the private-for-profit sector in the Eastern States, compared with 44% in the North and over 50% in West and South. These differences remain strong after adjustment. Finally, first births are slightly more likely to take place in private-for-profit facilities than other births.

## Discussion and Conclusions

The institutional birth rate in India is extremely low even for those living within easy reach (5 km) of a hospital. Unless the pace of change accelerates, it will take until 2025 for half of all rural births to be institutional and mid-century before 75% coverage is reached. The national goal of achieving 80% coverage by 2010 is extremely optimistic and results from the 2005-6 NFHS-3 show a continuation of the slow rise but no sign of acceleration [30]. Maternal health needs to become a political priority [31]. The results of this analysis (Table 1) show that institutional care seeking for child birth in rural India is currently influenced by community access, economic status, education, region and birth order. While education and region show the strongest associations, the focus of this paper is a comparison of the influences of access and economic status and results show the latter emerging as a more crucial determinant. The impact of high delivery costs and distance to services as barriers to care seeking was highlighted in the 2006 Lancet Maternal Survival Series [32,33].

This importance of economic factors shown by predicted probabilities in Table 1 confirms the pattern found previously in both the North and South [13,34-36]. A study in Maharashtra found that the average expenditure incurred per delivery was Rupees 512 (US$11.6). Amongst those in the lowest socio-economic group this ranged from Rs.160 (US$4.0) if it was a home delivery to Rs.230 (US$5.8) and Rs.1,039 (US$26.1) if the delivery had taken place in public or private-for-profit institutions, respectively. Cost was found to be critical in influencing the decision to seek care and the differential in the cost of private and public care was important in the choice of provider [12]. These figures, along with analysis in this paper demonstrating the influence of financial constraints on care seeking, provides support for the government policy to promote institutional delivery by providing cash transfers of US$17 to the poor. This transfer aims to cover travel and subsistence costs for pregnant women and their accompanying family members as well as the cost of care itself. Anecdotal evidence shows that it has been leading to an increase in institutional delivery [37]. India's growing prosperity should also accelerate progress. A further factor favouring increased use of obstetric services is fertility decline. A greater proportion of births will be first births, for whom institutional delivery is much more common than for subsequent births.

However, the importance of economic status should not be taken as grounds for dismissing the importance of geographical access. This can have a crucial influence on the second delay, delay in reaching an adequate health care facility, as cited in the "three delays" model [3]. Its significance has previously been demonstrated on a local level [18]. For example Stephenson & Tsui (2002) found that in Uttar Pradesh the presence of a secondary health facility increased care seeking for both pregnancy and childbirth [38]. However, the effect of access varies by state: a study focusing on rural Andhra Pradesh, Gujarat, Bihar and Rajastan found access to health services (measured by whether a hospital was available within 5 km of the village or not) to have a statistically significant effect only in Rajastan [36]. Using NFHS-2 data at the national level geographical access has previously been found to be a weakly significant determinant of institutionally delivery [35]. Previous analysis has used standard regression techniques, however, not taking into account the clustered nature of the data. The random-effects method used in this study, in which community level effects have been taken into consideration, finds a more significant association. It is expected however, that failure to account for clustering would overstate significance, and it is possible therefore that significance levels differ because of some other factor (sample selection, time period, etc).

It is important to note that this logistic approach with a village-level random effect does affect associations with socio-economic status as well as distance, making it necessary and valid. While communities will obviously share distance, they are also to a lesser extent likely to share wealth. As communities share distance more precisely it may be that effects of distance are diffused more than socio-economic status, but it is correct that this happens and it is probable that studies without such controls are giving unjustified emphasis to distance.

Physical proximity does not necessarily imply uptake, however. There is also no recognised definition of what constitutes reasonable access. If a 15 km criterion is used, then nearly two-thirds of rural Indians have access. This is an admittedly crude measure because it does not take into account the availability of motorised transport and roads. As noted earlier, the presence of a sealed road in each village was recorded in NFHS-2 but was found to have little influence on uptake of services. It has also been suggested that the influence of income and education would diminish as geographical access improves but interactions between distance and all other factors were tested for and none found. Expansion of services may therefore not be sufficient to promote utilisation. Even if there is latent demand for services, poor quality and high cost can inhibit utilisation. The absence of a relevant measure of quality was a limitation of this analysis. Reluctance to use institutional services may also be a problem with many mothers preferring to deliver at home even when services are affordable, accessible and of acceptable quality [39,40].

In India the public sector is perceived by many to be of low quality. The absence of even primary newborn care facilities, such as warming and resuscitation equipment, is common [41]. The private sector suffers different problems; there has been a proliferation of practitioners, some with no recognised medical qualification, but, despite the sometimes dubious quality of care, the seeking of private health care is a sign of wealth and status. Services in general need to be made more user-friendly, higher quality and the community mobilised to utilise them [40].

The other highly significant finding is the importance of the community context in determining the use of maternal health services. This probably reflects unobserved community-level social and cultural circumstances and service characteristics. Social interactions at this level may also have an effect, influencing people's attitudes and opinions regarding care seeking.

The very strong regional differences in place of delivery that exist even after adjustment for access, economic status, birth order and education suggest that there are further unexplained factors affecting perceived desirability or preference for institutional delivery in India. Demand for services is vital for utilisation to take place and, according to Chatterjee, it is created when permission and ability coincide. Education, is certainly influential in this as higher levels are often associated with greater autonomy [42].

Studies have found that the perceived need for care is sometimes much lower than bio-medically defined need. The belief that delivery is a natural process not requiring medical attention is thought to be particularly strong in the North [43]. The cost of services also varies regionally and hidden costs often in the form of under the counter payments inflate the cost of institutional delivery and act as a deterrent [12].

The results in Table 2 show that wealth is the strongest factor affecting the decision between a private-for profit or public facility amongst institutional births. After adjustment only the presence of higher education makes private delivery more likely. In the East the public sector is certainly much more heavily relied upon than in other regions, particularly the South. The East suffers from low availability of private services which may reflect greater discouragement of the private sector in West Bengal, the largest Eastern state, which for many years has been governed by a communist party. Results also suggest that heightened concern over first births is conducive to increasing demand for, and choice of, a private institution.

In India, areas very remote from services undoubtedly need better provision and in the shorter-term the outreach of skilled birth attendants (a component of RCH II) offers a compromise. However, in most areas the first priority is to increase demand and maximize utilization of existing services. Educational attainment, which generates demand, is slowly increasing but with low economic status so clearly also inhibiting use of services findings suggest that demand side financing, as is already being trialed by the government through cash payments to poor women, shows great potential for increasing rates of institutional delivery. Future analysis could usefully assist policy makers more directly in deciding where to place finite resources. This could involve exploring the comparative impact on institutional delivery of investing in either cash subsidies or the building of new facilities in underserved areas.

## Competing interests

The authors declare that they have no competing interests.

## Authors' contributions

This work formed part of AK's doctoral research. She carried out the data analysis and drafted the manuscript. JC supervised the thesis and was instrumental in designing the study and guiding the analysis, he made substantial comments and revisions to the draft. AS was on the advisory committee and provided particular advice and support to the statistical analysis. CR was also on the advisory committee and provided expertise on maternal health. All authors read and approved the final manuscript.

## Pre-publication history

The pre-publication history for this paper can be accessed here:

http://www.biomedcentral.com/1471-2393/10/30/prepub
